# Regional Arterial Infusion of Nafamostat Mesylate and Lidocaine in the Treatment of Cerulein-Induced Acute Pancreatitis in Rats

**DOI:** 10.5152/tjg.2025.24493

**Published:** 2025-09-10

**Authors:** Ryszard Antkowiak, Łukasz Antkowiak, Zbigniew Arent, Bogna Drozdzowska, Anna Kasperczuk, Jacek Bialecki, Agnieszka Pietsch-Fulbiszewska, Agata Cieslik-Bielecka, Pawel Domoslawski

**Affiliations:** 1 Department of General and Oncologic Surgery, St. Joseph Hospital, Mikolow, Poland; 2 Department of Neurosurgery, St. Barbara Regional Hospital and Trauma Center, Medical University of Silesia, Sosnowiec, Poland; 3 Experimental and Innovative Medicine Centre, University of Agriculture, Krakow, Poland; 4 Department of Pathomorphology, Medical University of Silesia Faculty of Medical Sciences, Zabrze, Poland; 5 Institute of Biomedical Engineering, Bialystok University of Technology Faculty of Mechanical Engineering, Bialystok, Poland; 6 Department of General Minimally Invasive and Trauma Surgery, Franciszek Raszeja Municipal Hospital, Poznan, Poland; 7 Department of Oral and Maxillofacial Surgery, St. Barbara Regional Hospital and Trauma Center, Sosnowiec, Poland; 8 Department of General Gastroenterologic and Endocrine Surgery, Wroclaw Medical University, Wroclaw, Poland

**Keywords:** Acute pancreatitis, infusions, intraarterial, microcirculation, protease inhibitors, vasodilation

## Abstract

**Background/Aims:**

The effectiveness of nafamostat for treating acute pancreatitis (AP) remains questionable. It was hypothesized that the administration of lidocaine would increase the penetration of nafamostat into the pancreas and improve its efficacy. This study evaluated the efficacy of combined management with nafamostat and lidocaine in the treatment of AP.

**Materials and Methods:**

Mild edematous AP was induced with cerulein in Wistar rats, which were distributed among 3 interventional cohorts: (1) a lidocaine cohort, with a regional intraarterial infusion of 1% lidocaine solution, (2) a lidocaine–nafamostat group, with a regional intraarterial infusion of 1% lidocaine solution followed by the infusion of nafamostat mesylate, and (3) an NaCl group, with a regional intraarterial infusion of 0.9% sodium chloride solution. Following 4, 8, and 12 hours after drug administration, serum amylase and lipase levels were tested. Rats were euthanized, and the pancreas was removed for histopathological examination.

**Results:**

A total of 16 rats were analyzed: 6 in the lidocaine group, 5 in the lidocaine–nafamostat group, and 5 in the NaCl group. Post-treatment amylase and lipase levels were comparable between the groups. The NaCl group had more prevalent signs of chronic inflammation in the pancreatic tissue and adipose tissue than both the lidocaine and lidocaine–nafamostat groups.

**Conclusion:**

The addition of nafamostat mesylate did not show superiority over lidocaine alone in the treatment of AP. Considering the prolonged observation period and self-healing tendency of mild edematous AP, both lidocaine and nafamostat do not impact treatment outcomes. However, both drugs may reduce the inflammatory and necrotic processes triggered by AP.

**Main Points**

- Nafamostat mesylate did not show superiority over lidocaine alone in treating mild edematous acute pancreatitis (AP).
- Nafamostat and lidocaine do not seem to influence the outcomes of mild edematous AP.
- Nafamostat and lidocaine may reduce the inflammatory and necrotic processes triggered by AP.

## Introduction

Acute pancreatitis (AP) is the most prevalent gastrointestinal disease. The incidence of AP is estimated at 34 cases per 100 000 person-years and is increasing constantly, and significant efforts have been made to establish effective treatments.^1^^,^^2^ Nevertheless, there is currently no effective treatment for AP apart from fluid therapy and analgesic administration.^3^ Microcirculatory disturbances play a crucial role in the pathogenesis of AP.^4^ A previous study induced vasodilation to specifically act on pancreatic microcirculation.^5^ It has been demonstrated that the course of AP was effectively improved by using 1% lidocaine solution administered through continuous regional arterial infusion (CRAI) into the celiac trunk. This improvement was reflected by pancreatic enzyme reduction and pathological examination of pancreatic specimens.^5^^,^^6^

Lidocaine acts as a vasodilator via voltage-gated sodium-channel blockage and cellular membrane stabilization.^7^ However, circulatory disturbances seem to result from initial proenzyme autoactivation with further secondary vasoconstriction and thrombotic complications. The continuous activation of proteases in AP leads to autodigestion of the pancreatic parenchyma, which results in an inflammatory response. The edema caused by the inflammatory process disturbs the pancreatic microcirculation, leading to a decrease in blood flow, which may result in the formation of microclots.^8^

Nafamostat is a synthetic low-molecular-weight protease inhibitor with anti-inflammatory and anticoagulant effects. It inhibits a wide range of proteases, including trypsin, thrombin, and plasmin, and it influences coagulation, the kallikrein–kinin system, and complement systems. Moreover, it has been shown to inhibit the production of pro-inflammatory cytokines.^4^ A recently published multicenter randomized controlled trial showed a lack of influence of nafamostat administered via the CRAI on preventing pancreatic necrosis.^9^ The administration of nafamostat should inhibit the activation of pancreatic proteases, reduce pancreatic and peripancreatic inflammation, and lead to a slight improvement in pancreatic microcirculation as a result of the anticoagulant properties of the drug.

It was hypothesized that the lack of therapeutic effect of nafamostat might result from insufficient drug penetration into the pancreatic microcirculation and the pancreatic parenchyma. Lidocaine dilates microcirculatory vessels in the pancreas, so it may allow better penetration of subsequently administered nafamostat into the pancreatic parenchyma. The targeted effect of nafamostat on the pathological mechanism underlying AP should then improve the course of the disease. Therefore, this study evaluated the efficacy of combined management with nafamostat and lidocaine compared with lidocaine alone for treating AP.

## Materials and Methods

### Ethical Approval

The study protocol was approved by the second Local Institutional Animal Care and Use Committee (IACUC) of the Institute of Pharmacology, Polish Academy of Sciences in Krakow (Approval no: 296/2019; Date: December 5, 2019) and in accordance with the Act of January 15, 2015 for the protection of animals used for scientific or educational purposes. The paper was prepared in compliance with the “Animal Research: Reporting of In Vivo Experiments” (ARRIVE) guidelines.^10^

### Study Cohort

This study was conducted on male albino Wistar rats at the Experimental and Innovative Medicine Center at the University of Agriculture in Krakow. Following ethical approval, the rats were collected from the Animal House of the Faculty of Pharmaceutics, Jagiellonian University Medical College in Krakow. The details of the animal care were described in detail in the previous study.^5^ The rats were distributed among 3 consecutive cohorts: (1) a control cohort (NaCl group), with a regional intraarterial infusion of 0.9% sodium chloride solution; (2) a lidocaine cohort, with a regional intraarterial infusion of 1% lidocaine solution (Lidocainum Hydrochloricum WZF 1%, Polfa Warszawa, Warsaw, Poland); and (3) a lidocaine–nafamostat cohort, with a regional intraarterial infusion of 1% lidocaine solution and then an infusion of nafamostat mesylate solution (Nafamostat mesylate, MCE, New Jersey, USA).

### Study Design

Initially, all animals had their blood tested for amylase and lipase levels using blood from the greater saphenous vein or caudal vein (measurement M0). In order to evoke mild edematous AP under experimental conditions, each animal was subcutaneously injected every hour with cerulein (Ceruletide, MCE, New Jersey, USA) at 20 µg/kg body weight in 4 doses (total dose of 80 µg/kg body weight). At 6 hours after the final injection of the cerulein solution (10 hours following the first cerulein injection), blood tests were performed again (measurement M1) to determine the serum amylase and lipase levels at the time of active AP. Shortly after, a surgical procedure was performed.

A detailed description of the surgical technique and celiac trunk approach is provided in a previous paper.^5^ In order to achieve intraarterial drug infusion, abdominal aorta was punctured at the point where celiac trunk branches off, directly with a 26G venous cannula. All intraarterial infusions were performed slowly. In the NaCl group, 5 mg/kg body weight of 0.9% NaCl solution was administered in a bolus. Rats from the lidocaine group, received a 5 mg/kg body weight bolus of lidocaine. In lidocaine–nafamostat group, a 5 mg/kg body weight bolus of lidocaine was administered, followed 1 minute by a 6 mg/kg body weight bolus of nafamostat mesylate.

After hemostasis was achieved, the surgical procedure was terminated. Finally, blood tests were performed at 4 hours (measurement M2), 8 hours (measurement M3), and 12 hours (measurement M4) after the surgery. Following the last blood test, the euthanasia of each animal was performed using pentobarbital at 300 mg/kg body weight (Morbital, Biowet Pulawy, Pulawy, Poland). Pancreatic tissue and surrounding adipose tissue were collected for postmortem histopathological examination.

### Histopathological Examination

Postmortem specimens of pancreatic tissue and peripancreatic adipose tissue were preserved in 10% formaldehyde. Preprepared paraffin blocks were cut and dyed in hematoxylin and eosin. The histopathological examination was conducted by a pathologist (B.D.) unaware of subjects’ distribution into cohorts. The presence and severity of necrosis, edema, hemorrhagic foci, acute inflammatory response, and chronic inflammatory response was evaluated in the pancreatic tissue. Moreover, the presence and severity of necrosis, hemorrhagic foci, acute inflammatory response, and chronic inflammatory response was assessed in the adipose tissue. Each pancreas was additionally assessed for the quantity of veins, arteries, and capillaries. The severity of abovementioned features was evaluated using the previously published scale: 0—feature not observed; 1—mild changes; 2—moderate changes; and 3—severe changes.^11^^–^^13^

### Statistical Analysis

Intergroup differences in amylase and lipase levels, the number of arteries and veins, and the number of capillaries were evaluated with the Mann‒Whitney *U* test. The Pearson chi-squared test was used to test the correlation between treatment type and histopathological findings. Values of *P* < .05 were considered statistically significant. All analyses were performed using Statistica 13.3 (StatSoft Polska; Krakow, Poland).

## Results

### Pancreatic Enzymes

**Figure 1.**
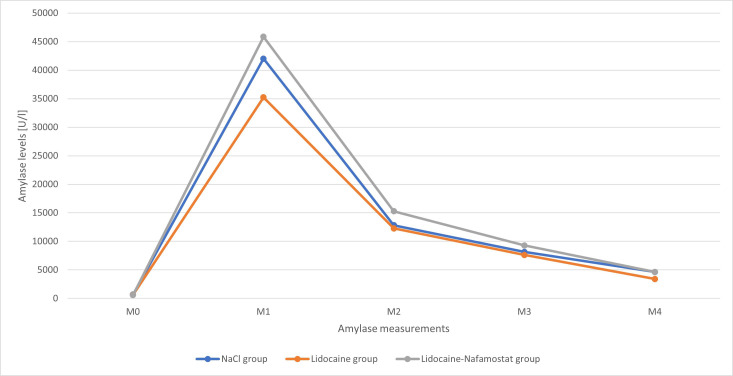
Amylase levels in the NaCl group, lidocaine group, and lidocaine–nafamostat group over the entire preoperative (M0-M1) and postoperative (M2-M4) course. M0—initial amylase levels, M1—amylase levels after the induction of pancreatitis just before the operation, M2—amylase levels 4 hours after drug administration, M3—amylase levels 8 hours after drug administration, M4—amylase levels 12 hours after drug administration.

**Figure 2.**
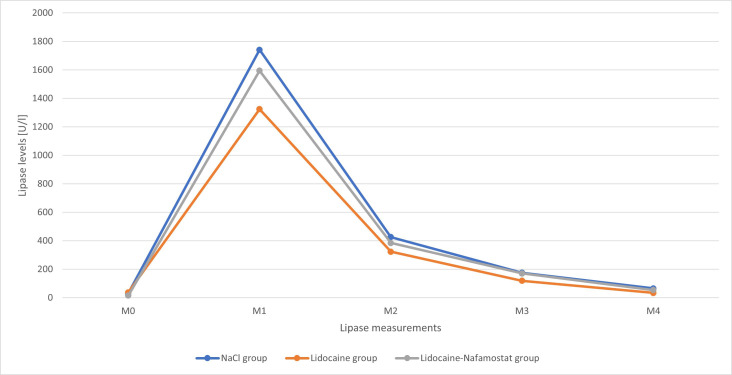
Lipase levels in the NaCl group, lidocaine group, and lidocaine–nafamostat group over the entire preoperative (M0-M1) and postoperative (M2-M4) course. M0—initial lipase levels, M1—lipase levels after the induction of pancreatitis just before the operation, M2—lipase levels 4 hours after drug administration, M3—lipase levels 8 hours after drug administration, M4—lipase levels 12 hours after drug administration.

A total of 16 rats met the inclusion criteria and were further analyzed. There were 5 rats in the NaCl group, 6 rats in the lidocaine group, and 5 rats in the lidocaine–nafamostat group. The baseline amylase levels (M0) were comparable between all groups. In contrast, significantly higher lipase levels were noted in the lidocaine group than in the lidocaine–nafamostat group (mean values of 37 U/l and 15 U/l, respectively; *P* = .018). Lipase and amylase levels measured after induction of AP (M1) and those demonstrating disease dynamics after treatment administration (M2-M4) were comparable between all 3 groups (Tables 1 and 2). The time-dependent changes in amylase and lipase levels are illustrated in Figures 1 and 2.

### Histopathology

Histopathological specimens were available for 5 of the 6 rats (83%) in the lidocaine group and all rats in the NaCl group (100%) and lidocaine–nafamostat group (100%). The exclusion of 1 specimen in the lidocaine group resulted from a technical issue that precluded reliable histopathological analysis. According to the analysis, the NaCl group had a significantly higher rate of chronic inflammation of the pancreatic tissue compared to both the lidocaine group (*P* = .038) and the lidocaine–nafamostat group (*P* = .010). No intergroup differences were observed in terms of necrosis, edema, and acute inflammation of the pancreatic tissue, as well as the presence of hemorrhagic foci in both the pancreatic tissue and adipose tissue. There were no signs of necrosis or acute inflammation of the adipose tissue. All evaluated features present were considered mild (grade 1) except for 1 pancreatic tissue specimen from the NaCl group, which showed a moderate level of edema (grade 2).

The quantitative analysis revealed a significantly higher number of veins and arteries in the NaCl group compared to both the lidocaine group (*P* = .037) and the lidocaine–nafamostat group (*P* = .022). Moreover, the mean number of capillaries was significantly higher in the NaCl group than in the lidocaine–nafamostat group (*P* = .047), while no significant differences were observed between the NaCl group and the lidocaine group, as well as between the lidocaine group and the lidocaine–nafamostat group. The detailed results of the histopathological examination are presented in Table 3.

## Discussion

Induction of an inflammatory cascade with inappropriate and excessive activation of proteases in AP leads to microcirculatory disturbances within the pancreatic tissue.^14^ When an AP-triggering factor occurs, it converts trypsinogen to trypsin in acinar cells, resulting in their damage and the release of enzymes into the pancreatic parenchyma. Subsequent autolysis of the pancreatic tissue and its vessels with concurrent activation of other pancreatic enzymes increases pancreatic vascular permeability, leading to pancreatic tissue edema and thus secondary microcirculatory disturbances. In AP, vasoconstriction and enhanced intravascular coagulation increase ischemia, which impairs the penetration of drugs into the pancreatic tissue.^15^^,^^16^ Local vasoconstriction makes it challenging to achieve high concentrations of intravenously administered medications in pancreatic tissue. Therefore, considering the AP cascade, it seems to be a reasonable approach to treat AP through vasodilatation to facilitate drug administration and prevent further necrotic changes in pancreatic tissue.

Thus, previous experimental studies on cerulein-induced AP in rats have also evaluated the vasodilatory effect of CRAI of 1% lidocaine solution on pancreatic arterial circulation.^5^^,^^6^ Lidocaine is widely used in microsurgery as a safe and effective vasodilator,^17^^-^^19^ but the exact mechanism has not been fully elucidated. Notably, the effect is concentration-dependent, with lower doses causing vasoconstriction.^7^ It has been shown that lidocaine stabilizes the cell membrane and calcium inflow and blocks voltage-gated sodium channels.^7^ Considering these effects, it has been hypothesized that 1% lidocaine might dilate the arterial vascular system in pancreatic tissue, which improves the course of AP.

Consequently, it has been found that lidocaine meaningfully impacts the course of AP, which was reflected by significantly lower pancreatic enzyme levels in the lidocaine group than in the control group.^5^ Additionally, amylase and lipase levels were compared between the treatment endpoint (M5) and shortly after AP induction (M2). The lidocaine group exhibited significantly greater decreases in amylase (79%) and lipase (87%) levels than the control group, in which amylase and lipase decreased by 60% and 70%, respectively.^5^ Moreover, a recently published experimental study revealed superior efficacy of intraarterial infusion of 1% lidocaine solution compared to low-molecular-weight heparin.^6^

Considering the cascade of pathological response in AP, an aim of the present study was to determine whether adding the protease inhibitor nafamostat to lidocaine would improve the course of AP. Nafamostat affects pancreatic enzyme activation and also inhibits phospholipase A2, the complement, and fibrinolytic systems, including thrombin, kallikrein, plasmin, and factors VII and IX. Moreover, it inhibits the production of pro-inflammatory cytokines, reactive oxygen species, and proteolytic enzymes.^9^^,^^20^^,^^21^ Notably, in AP, thrombin stimulates the production of endothelin, which is known to constrict the pancreatic microcirculation, leading to the formation of necrotic foci through intravascular coagulation.^9^^,^^20^

Therefore, it was hypothesized that pretreatment with lidocaine would dilate the pancreatic microcirculation, thus improving the penetration of nafamostat into the pancreas. Additionally, it is believed that highly concentrated nafamostat mesylate inhibits pancreatic enzyme autoactivation and reduces endothelin-mediated vasospasm and associated thrombotic events, eventually enhancing the therapeutic effect of lidocaine alone. However, the study showed that the CRAI of lidocaine and subsequent infusion of nafamostat mesylate into the celiac trunk was not superior to CRAI of lidocaine into the celiac trunk alone. Furthermore, both lidocaine and the combined treatment of lidocaine with nafamostat did not show superior efficacy over control group.

The results of the present study are somewhat contradictory to 2 previously published studies, which showed significant efficacy of lidocaine in AP. However, both studies that evaluated the course of mild edematous AP by assessing the pancreatic enzyme levels after 1, 3, and 5 hours following initiation of AP treatment, showed significant impact of lidocaine on the course of AP.^5^^,^^6^ In the present study, pancreatic enzyme levels were evaluated after 4, 8, and 12 hours. All of these studies revealed that pancreatic enzymes decreased naturally with time, even in the control groups. Considering the self-healing tendency of mild edematous AP, the prolonged observation of pancreatic enzyme dynamics might not have been sensitive enough to pick up subtle changes in the course of AP, which is influenced by lidocaine alone and by combined treatment with lidocaine and nafamostat. Therefore, further studies on severe AP model are warranted to define the influence of either lidocaine or nafamostat on treatment outcomes.

Nevertheless, the study showed that both lidocaine and combined treatment with lidocaine and nafamostat significantly reduced the inflammatory processes and necrosis in pancreatic and adipose tissue. Therefore, it was hypothesized that lidocaine and nafamostat may influence the course of AP, while their impact on final outcomes has yet to be determined. Mikami et al^22^ examined a rat model of severe AP induced by 5% sodium taurocholate. They reported that the CRAI of nafamostat mesylate significantly reduced serum levels of interleukin 6 (IL-6) and trypsinogen activation peptide compared to those in untreated controls. Moreover, histological examination revealed decreased pancreatic necrosis in rats receiving nafamostat infusion. Similarly, Keck et al^23^ highlighted the anti-inflammatory effect of nafamostat in a rat model of cerulein-induced severe necrotizing AP. Furthermore, some authors have reported a positive impact of nafamostat on the course of AP in a clinical setting. Horibe et al^21^ performed a meta-analysis of 8 observational studies and reported that CRAI with protease inhibitors resulted in a lower mortality rate and lower risk for surgical intervention. In contrast, in a multicenter study of 1097 patients, Horibe et al^24^ observed no impact of CRAI with protease inhibitors on the mortality rate and the need for surgical intervention. Similarly, using nationwide data from 414 patients, Hamada et al^25^ found that nafamostat administered via CRAI did not reduce the mortality rate, and this approach significantly increased the risk of interventions due to complications of infection.

The histopathological findings showed a significantly lower number of capillaries in the lidocaine–nafamostat group compared to the NaCl group, as well as a significantly lower number of veins and arteries in both study groups compared to the NaCl group. While the number of veins and arteries seems unrelated to drug administration, since none of these drugs act on such vessels, it is surprising that the NaCl group had a significantly higher number of capillaries, which are the target vessels for vasodilatory drugs such as lidocaine. However, the type of estimation used is far from perfect, and researchers are encouraged to investigate the impact of vasoactive drugs further via Doppler ultrasonography.

Despite the potential benefits, prolonged intraoperative bleeding from the mesentery was observed in all rats receiving nafamostat mesylate infusion. In contrast, no spontaneous intraoperative bleeding episodes were found in rats that received only the lidocaine infusion. Similar to the findings, some authors have also reported hemorrhagic complications following nafamostat mesylate infusion.^21^^,^^26^ However, nafamostat was infused at 6 mg/kg (420 mg for a standard 70-kg patient) for 1-2 minutes. In a clinical setting, this drug is infused at 120-336 mg over a much longer period of 3-7 days.^21^ Therefore, it is believed that the extremely short period of nafamostat infusion together with the high dosage might have led to the high rate of intraoperative bleeding. Histopathologically, neither the pancreatic nor the adipose tissues in the study showed signs of hemorrhage.

Despite the novelty in the design of the experimental study, there are some significant limitations that must be highlighted. Firstly, the study was based on only 16 rats, which could have significantly influenced the strength of the conclusions. Moreover, the expanded blood-test time points at 4, 8, and 12 hours after the AP treatment procedure may have significantly influenced the results due to fast changes being overlooked within the shorter observation time.

Considering this together with the induction of mild edematous AP (instead of severe AP), as well as the natural healing tendency of all rats (reflected by a gradual decrease of pancreatic enzymes in all groups over time, including NaCl group), no solid conclusions on the impact of tested drugs on outcomes can be made. Moreover, nafamostat is typically administered via longer-lasting infusion, so the short-lasting infusion might have influenced the efficacy of the drug in the experimental model of AP. Therefore, despite this study being the first to determine the effects of combined treatment with lidocaine and nafamostat in AP, researchers are encouraged to perform larger experimental studies on the topic, preferably with longer nafamostat infusion.

The addition of nafamostat mesylate did not show any superiority over lidocaine alone in the treatment of AP. Considering the prolonged observation period and self-healing tendency of mild edematous AP in rats, both lidocaine and nafamostat do not impact outcomes of edematous AP. Both lidocaine and nafamostat may reduce the inflammatory and necrotic processes triggered by AP, thus improving the course of AP. Further studies on severe AP are warranted to determine the influences of lidocaine and nafamostat on treatment outcomes.

## Figures and Tables

**Table 1.** Comparison of Amylase Levels Between the NaCl, Lidocaine, and Lidocaine–Nafamostat Groups over the Whole Course of the Study

Measurement	NaCl Group, (U/l)	Lidocaine Group, (U/l)	Lidocaine–Nafamostat Group, (U/l)	*P*
M0	662	626	587	*.201; **.095; ***.315
M1	42023	35229	45848	*.235; **.210; ***.171
M2	12820	12272	15282.5	*.411, **.296; ***.523
M3	8145	7613	9287	*.648, **.676; ***.411
M4	4642	3405	4632	*.315, **.835; ***.315

*Values for comparison of amylase levels between NaCl and lidocaine group.

**Values for comparison of amylase levels between NaCl and lidocaine–nafamostat group.

***Values for comparison of amylase levels between Lidocaine and lidocaine–nafamostat group.

**Table 2.** Comparison of Lipase Levels Between the NaCl Group, Lidocaine, and Lidocaine–Nafamostat Groups over the Whole Course of the Study

Measurement	NaCl Group, [U]	Lidocaine Group, [U]	Lidocaine–Nafamostat Group, [U]	*P*
M0	30	37	15	*.411; **.296; ***.018
M1	1741	1324	1595	*.121; **1.000; ***.315
M2	426	323	385	*.927; **.676; ***.171
M3	175	119	171	*.171; **.531; ***.235
M4	65	34	54	*.083; **.210; ***.784

*Values for comparison of lipase levels between NaCl and lidocaine group.

**Values for comparison of lipase levels between NaCl and lidocaine–nafamostat group.

***Values for comparison of lipase levels between lidocaine and lidocaine–nafamostat group.

**Table 3.** Comparison of Histopathological Findings Between the NaCl Group, Lidocaine, and the Lidocaine–Nafamostat Groups

Feature	NaCl Group	Lidocaine Group	Lidocaine–Nafamostat Group	*P*
Pancreatic tissue				
Necrosis	1/5 (20%)	2/5 (40%)	0/5 (0%)	*.490; **.292; ***.114
Edema	2/5 (40%)	0/5 (0%)	0/5 (0%)	*.287; **.287; ***-
Hemorrhagic foci	1/5 (20%)	0/5 (0%)	0/5 (0%)	*.292; **.292; ***-
Acute inflammation	1/5 (20%)	0/5 (0%)	0/5 (0%)	*.292; **.292; ***-
Chronic inflammation	5/5 (100%)	2/5 (40%)	1/5 (20%)	*.038; **.010; ***0.490
Mean number of veins and arteries, SD	14.6 (3.21)	7.8 (3.83)	5.8 (3.9)	*.037; **.022; ***.458
Mean number of capillaries, SD	30.0 (7.07)	20.6 (9.34)	16.6 (9.53)	*.095; **.047; ***.463
Adipose tissue				
Necrosis	0/5 (0%)	0/5 (0%)	0/5 (0%)	*-; **-; ***-
Hemorrhagic foci	1/5 (20%)	0/5 (0%)	0/5 (0%)	*.292; **.292; ***-
Acute inflammation	0/5 (0%)	0/5 (0%)	0/5 (0%)	*-; **-; ***-
Chronic inflammation	4/5 (80%)	0/5 (0%)	0/5 (0%)	*.010; **.010; ***-

*Values for comparison of features between NaCl and lidocaine group.

**Values for comparison of features between NaCl and Lidocaine–Nafamostat group.

***Values for comparison of features between Lidocaine and Lidocaine–Nafamostat group.

## Data Availability

The analyzed data sets generated during the present study are available from the corresponding author upon reasonable request.

## References

[1] Boxhoorn L Voermans RP Bouwense SA Acute pancreatitis. Lancet. 2020;396(10252):726 734. (doi: 10.1016/S0140-6736(20)31310-6) 32891214

[2] Lee PJ Papachristou GI. New insights into acute pancreatitis. Nat Rev Gastroenterol Hepatol. 2019;16(8):479 496. (doi: 10.1038/s41575-019-0158-2) 31138897

[3] Mao E. Intensive management of severe acute pancreatitis. Ann Transl Med. 2019;7(22):687 687. (doi: 10.21037/atm.2019.10.58) 31930088 PMC6944592

[4] Antkowiak R Bialecki J Chabowski M Domoslawski P. Treatment of microcirculatory disturbances in acute pancreatitis: where are we now? Pancreas. 2022;51(5):415 421. (doi: 10.1097/MPA.0000000000002044) 35973016

[5] Antkowiak R Antkowiak Ł Grzegorczyn S Efficacy of intra-arterial lidocaine infusion in the treatment of cerulein-induced acute pancreatitis. Adv Clin Exp Med. 2020;29(5):587 595. (doi: 10.17219/acem/121932) 32459401

[6] Antkowiak R Antkowiak L Arent Z Intraarterial infusion of lidocaine is superior to the subcutaneous injection of low molecular weight heparin for improving the course of cerulein-induced acute pancreatitis in rats. Arch Immunol Ther Exp (Warsz). 2025;73(1). (doi: 10.2478/aite-2025-0012) 40237149

[7] Ogawa H Kusumoto J Nomura T Hashikawa K Terashi H Sakakibara S. Wire myography for continuous estimation of the optimal concentration of topical lidocaine as a vasodilator in microsurgery. J Reconstr Microsurg. 2021;37(6):541 550. (doi: 10.1055/s-0040-1722759) 33517569

[8] Vollmar B Menger MD. Microcirculatory dysfunction in acute pancreatitis. A new concept of pathogenesis involving vasomotion-associated arteriolar constriction and dilation. Pancreatology. 2003;3(3):181 190. (doi: 10.1159/000070727) 12817573

[9] Hirota M Shimosegawa T Kitamura K Continuous regional arterial infusion versus intravenous administration of the protease inhibitor nafamostat mesilate for predicted severe acute pancreatitis: a multicenter, randomized, open-label, phase 2 trial. J Gastroenterol. 2020;55(3):342 352. (doi: 10.1007/s00535-019-01644-z) 31758329 PMC7026212

[10] Percie du Sert N Hurst V Ahluwalia A The ARRIVE guidelines 2.0: updated guidelines for reporting animal research. PLoS Biol. 2020;18(7):e3000410. (doi: 10.1371/journal.pbio.3000410) PMC7360023 32663219

[11] Zhang J Rouse RL. Histopathology and pathogenesis of caerulein-, duct ligation-, and arginine-induced acute pancreatitis in Sprague-Dawley rats and C57BL6 mice. Histol Histopathol. 2014;29(9):1135 1152. (doi: 10.14670/HH-29.1135) 24585404

[12] Tomaszewska R Dembiński A Warzecha Z Ceranowicz P Stachura J. Morphological changes and morphological-functional correlations in acute experimental ischemia/reperfusion pancreatitis in rats. Pol J Pathol. 2000;51(4):179 184. 11247388

[13] Bukowczan J Warzecha Z Ceranowicz P Kuśnierz-Cabala B Tomaszewska R. Obestatin accelerates the recovery in the course of ischemia/reperfusion-induced acute pancreatitis in rats. PLoS One. 2015;10(7):e0134380. (doi: 10.1371/journal.pone.0134380) PMC4520493 26226277

[14] Cuthbertson CM Christophi C. Disturbances of the microcirculation in acute pancreatitis. Br J Surg. 2006;93(5):518 530. (doi: 10.1002/bjs.5316) 16607683

[15] Eibl G Buhr HJ Foitzik T. Therapy of microcirculatory disorders in severe acute pancreatitis: what mediators should we block? Intensive Care Med. 2002;28(2):139 146. (doi: 10.1007/s00134-001-1194-1) 11907656

[16] Bhatia M Wong FL Cao Y Pathophysiology of acute pancreatitis. Pancreatology. 2005;5(2-3):132 144. (doi: 10.1159/000085265) 15849484

[17] Vargas CR Iorio ML Lee BT. A systematic review of topical vasodilators for the treatment of intraoperative vasospasm in reconstructive microsurgery. Plast Reconstr Surg. 2015;136(2):411 422. (doi: 10.1097/PRS.0000000000001431) 25909299

[18] Rinkinen J Halvorson EG. Topical vasodilators in microsurgery: what is the evidence? J Reconstr Microsurg. 2017;33(1):1 7. (doi: 10.1055/s-0036-1592191) 27636540

[19] Ricci JA Koolen PG Shah J Tobias AM Lee BT Lin SJ. Comparing the outcomes of different agents to treat vasospasm at microsurgical anastomosis during the papaverine shortage. Plast Reconstr Surg. 2016;138(3):401e 408e. (doi: 10.1097/PRS.0000000000002430) 27556614

[20] Piaścik M Rydzewska G Milewski J The results of severe acute pancreatitis treatment with continuous regional arterial infusion of protease inhibitor and antibiotic: a randomized controlled study. Pancreas. 2010;39(6):863 867. (doi: 10.1097/MPA.0b013e3181d37239) 20431422

[21] Horibe M Egi M Sasaki M Sanui M. Continuous regional arterial infusion of protease inhibitors for treatment of severe acute pancreatitis: systematic review and meta-analysis. Pancreas. 2015;44(7):1017 1023. (doi: 10.1097/MPA.0000000000000375) 26355545

[22] Mikami Y Takeda K Matsuda K Rat experimental model of continuous regional arterial infusion of protease inhibitor and its effects on severe acute pancreatitis. Pancreas. 2005;30(3):248 253. (doi: 10.1097/01.mpa.0000153328.54569.28) 15782103

[23] Keck T Balcom JH Antoniu BA Lewandrowski K Warshaw AL Fernández-del Castillo CF. Regional effects of nafamostat, a novel potent protease and complement inhibitor, on severe necrotizing pancreatitis. Surgery. 2001;130(2):175 181. (doi: 10.1067/msy.2001.115827) 11490346

[24] Horibe M Sasaki M Sanui M Continuous regional arterial infusion of protease inhibitors has no efficacy in the treatment of severe acute pancreatitis: a retrospective multicenter cohort study. Pancreas. 2017;46(4):510 517. (doi: 10.1097/MPA.0000000000000775) 27977624 PMC5359786

[25] Hamada T Yasunaga H Nakai Y Continuous regional arterial infusion for acute pancreatitis: a propensity score analysis using a nationwide administrative database. Crit Care. 2013;17(5):R214. (doi: 10.1186/cc13029) PMC4055985 24088324

[26] Takeda K Matsuno S Sunamura M Kakugawa Y. Continuous regional arterial infusion of protease inhibitor and antibiotics in acute necrotizing pancreatitis. Am J Surg. 1996;171(4):394 398. (doi: 10.1016/S0002-9610(97)89617-1) 8604829

